# Cystatin C Has a Dual Role in Post-Traumatic Brain Injury Recovery

**DOI:** 10.3390/ijms15045807

**Published:** 2014-04-04

**Authors:** Marina Martinez-Vargas, Maribel Soto-Nuñez, Erika Tabla-Ramon, Barbara Solis, Ruben Gonzalez-Rivera, Adan Perez-Arredondo, Francisco Estrada-Rojo, Andres Castell, Juan Molina-Guarneros, Luz Navarro

**Affiliations:** 1 Department of Physiology, School of Medicine, Universidad Nacional Autonoma de Mexico, Apdo. Postal 70-250, Mexico, D.F. 04510, Mexico; E-Mails: marvar_69@yahoo.com.mx (M.M.-V.); erimar_1503@yahoo.com.mx (E.T.-R.); bsolluna@hotmail.com (B.S.); rubengonzalezrivera@yahoo.com.mx (R.G.-R.); aparredondo23@yahoo.com.mx (A.P.-A.); fesro@hotmail.com (F.E.-R.); 2 Department of Pharmacology, School of Medicine, Universidad Nacional Autonoma de Mexico, Apdo. Postal 70-250, Mexico, D.F. 04510, Mexico; E-Mails: inmunofarmacologia@yahoo.com (M.S.-N.); jamg@unam.mx (J.M.-G.); 3 Department of Cell and Tissue Biology, School of Medicine Universidad Nacional Autonoma de Mexico, Apdo. Postal 70-250, Mexico, D.F. 04510, Mexico; E-Mail: castell@unam.mx

**Keywords:** Cystatin C, Cathepsin B, Traumatic brain injury, neuroprotection

## Abstract

Cathepsin B is one of the major lysosomal cysteine proteases involved in neuronal protein catabolism. This cathepsin is released after traumatic injury and increases neuronal death; however, release of cystatin C, a cathepsin inhibitor, appears to be a self-protective brain response. Here we describe the effect of cystatin C intracerebroventricular administration in rats prior to inducing a traumatic brain injury. We observed that cystatin C injection caused a dual response in post-traumatic brain injury recovery: higher doses (350 fmoles) increased bleeding and mortality, whereas lower doses (3.5 to 35 fmoles) decreased bleeding, neuronal damage and mortality. We also analyzed the expression of cathepsin B and cystatin C in the brains of control rats and of rats after a traumatic brain injury. Cathepsin B was detected in the brain stem, cerebellum, hippocampus and cerebral cortex of control rats. Cystatin C was localized to the choroid plexus, brain stem and cerebellum of control rats. Twenty-four hours after traumatic brain injury, we observed changes in both the expression and localization of both proteins in the cerebral cortex, hippocampus and brain stem. An early increase and intralysosomal expression of cystatin C after brain injury was associated with reduced neuronal damage.

## 1. Introduction

Traumatic brain injury (TBI) is one of the most important health problems in the world today. It is the leading cause of brain damage in children and young adults, and in Europe, it is responsible for longer periods of disability than any other cause [1].

In the United States, more than 1.7 million individuals suffer a TBI annually, resulting in 52,000 deaths [2].

A TBI triggers pathological pathways that may potentially harm brain cells. These mechanisms include excitotoxicity, the formation of free radicals and the release of lysosomal proteases. However, self-protective mechanisms are also induced by brain injury. These mediators have damage-reducing properties and are endogenous efforts to counteract traumatic damage and improve neuronal repair [3]. The balance between the harming and the protective mechanisms will ultimately determine the fate of the injured brain [3]. For instance, cathepsins are lysosomal cystein-proteases that appear to be released after traumatic injury and increase neuronal death; on the other hand, the release of cystatin C (CC), an endogenous inhibitor of cathepsins B, H, K, L, and S, appears to be a self-protective brain response [4].

Cathepsin B is one of the major lysosomal cysteine proteases involved in protein catabolism in neurons, and it also plays an important role in necrotic and apoptotic programmed cell death [5,6]. An increase in the cathepsin B enzymatic activity and/or immunoreactivity in TBI models in rats or mice has been reported [7–9]. A lysosomal-mitochondrial axis theory of cell-death proposes that cathepsin B activates BH3 interacting-domain death agonist (Bid) through lysosome–mitochondria cross signaling and either directly or indirectly activates the Bax/Bak-dependent caspase cascade leading to apoptosis [10]. It also has been described that in cathepsin B knockout mice there is a considerable amelioration of damage by using a controlled cortical impact TBI model [8]. In addition, pretreatment with specific inhibitors of cathepsin B remarkably attenuates cell death, lesion volume and motor and cognitive dysfunction in a model of TBI in mice [9]. Furthermore, treatment with inhibitors of cathepsin B is effective even when administered eight hours after injury [8]. Nevertheless, we found that intracerebroventricular administration of a high dose of CC worsened TBI recovery [11], despite being one of the most potent endogenous inhibitors of cathepsin B [12], and decreased the central temperature [13]. Considering all of these data, our study aimed to determine the effects of lower doses of CC during rat TBI recuperation. We also analyzed the time course of the expression of cystatin C and cathepsin B in the brain of rats after a TBI.

## 2. Results and Discussion

### 2.1. Intracerebroventricular Injection of Cystatin C

**Figure 1.**
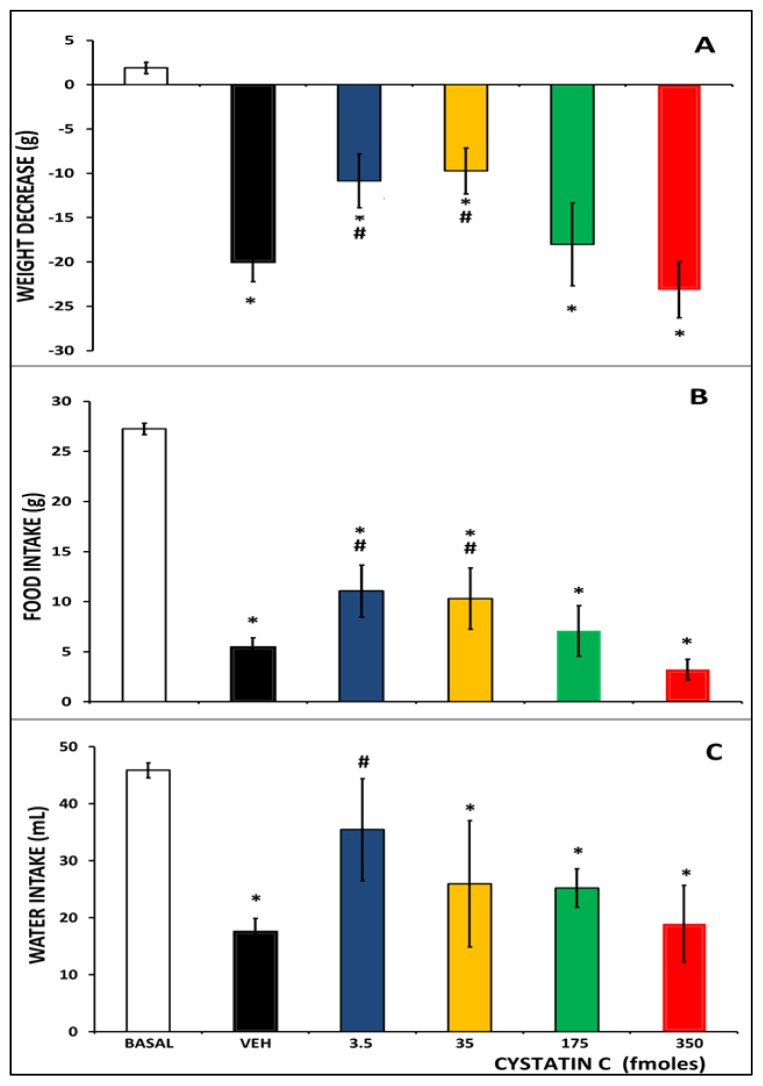
Effect of CC administration before TBI on weight decrease, food and water intake. (**A**) Bars represent mean ± SEM of weight (g) measured 24 h after TBI; (**B**) Bars represent mean ± SEM of food intake (g) 24 h after TBI; (**C**) Bars represent mean ± SEM of water intake (mL) 24 h after TBI. * *p* < 0.05 *vs*. basal, # *p* < 0.05 *vs.* veh; One-way ANOVA and Duncan’s test as *post hoc*.

We observed significant differences in both the body weight (*F*_5,118_ = 39.35, *p* < 0.0001) and the food (*F*_5,133_ = 107.54, *p* < 0.0001) and water (*F*_5,92_ = 22.34, *p* < 0.0001) intake 24 h after TBI in all experimental groups compared to the control rats (see Figure 1). Administration of either 3.5 or 35 fmoles of CC before TBI produced a lesser decrease in body weight and in food intake compared with the vehicle or the 350 fmoles groups (see Figure 1A,B). In addition, we observed a statistically significant increase in water intake in the rats treated with 3.5 fmoles of CC compared to rats treated with vehicle or 350 fmoles CC before TBI (see Figure 1C). TBI impairs the physical state of the rat, which manifests itself through decreased body weight and decreased intake of food and water. These changes are part of the metabolic response to trauma [14], and they have been used by other researchers to evaluate the neuroprotective effects of various substances in brain injury models [15]. We found that the administration of low doses of CC reduced this impairment, which is in agreement with effect of cystatin C in control rats. Higher doses of cystatin C reduced food intake and body weight whereas lower doses increased them (data not shown).

**Figure 2.**
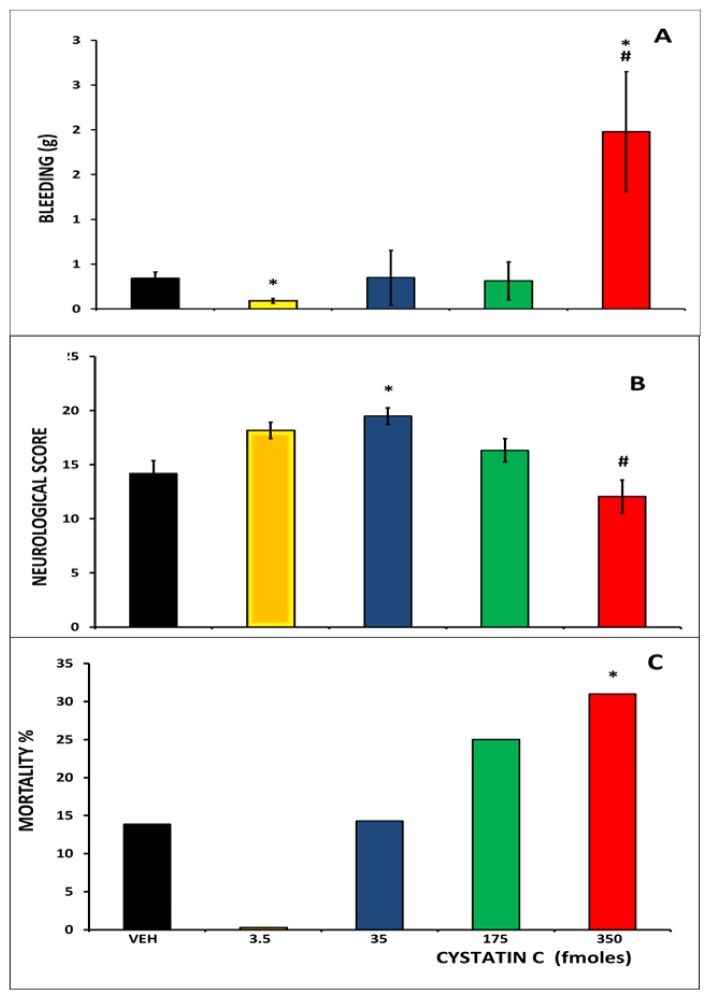
Effect of CC administration before TBI on bleeding, neurological score and mortality. (**A**) Bars represent mean ± SEM of bleeding (g) measured 15 min after TBI. * *p* < 0.05 *vs.* veh, # *p* < 0.05 *vs*. all other groups; Kruskall Wallis and Mann Whitney test; (**B**) Bars represent mean ± SEM of neurological scores obtained 24 h after TBI. * *p* < 0.05 *vs.* veh, # *p* < 0.05 *vs.* CC 35; Kruskall Wallis and Mann Whitney test; (**C**) Bars represent the mortality percentage eight days after TBI. * *p* < 0.05, Chi square test.

We also analyzed the effects on survival and recovery of the intracerebroventricular injection of CC at different doses (see Figure 2). We observed statistically significant differences between the level of bleeding caused by the TBI in the vehicle and CC groups (KW = 21.76, *p* < 0.001): the lowest doses (3.5 fmoles) decreased bleeding, whereas the highest doses (350 fmoles) increased it (see Figure 2A). Furthermore, the lowest dose of CC reduced the bleeding provoked by TBI, whereas the highest dose increased this bleeding; these data are consistent with previous reports from our group [11]. The highest dose of CC also increased the mortality rate, whereas no deaths were observed at the lowest dose.

We also observed statistically significant differences between the neurological scores of rats 24 h after TBI in CC administered rats *vs.* the vehicle group rats (KW = 13.52, *p* < 0.009). Intracerebroventricular injection of 35 fmoles increased the score on the neurobehavioral test compared to rats injected with vehicle or with 350 fmoles of CC before TBI (see Figure 2B). We observed better scores for the neurological damage tests with low doses, but we did not observe significant changes between the larger doses and vehicle, which we attribute to the fact that the corresponding tests could only be performed in surviving rats.

We also observed a significant increase in the mortality eight days after TBI in rats injected with 350 fmoles of CC before TBI compared to all the other groups (chi-square > 4.20, 1 d.f.; *p* < 0.05; see Figure 2C). The lowest dose of CC had an apparent decrease in mortality compared to the vehicle, but it was not statistically significant.

There are contradictory reports about the role of CC as a neuroprotector, possibly due to the doses used. For example, the inhibition of cathepsin B and L by CC seems to protect CA1 after global ischemia in primates [16], and specific inhibitors of cathepsin B remarkably attenuates the damage in a model of TBI in mice [8,9], whereas intrahippocampal administration of CC in rats caused neuronal loss in the dentate granular cell layer [17]. This effect is most likely associated with the inhibition of cathepsin B, as a later study by Nagai [18] found that coadministration of CC with cathepsin B attenuated the damage caused by hippocampal administration; moreover, in the same report, Nagai found a dose-dependent response for the cell survival reduction in cultures of human neurons, whereas Olson *et al.* [19] have shown that gene deletion of CC aggravates brain damage following focal ischemia but protects against it after global ischemia.

### 2.2. Basal Distribution of Cathepsin B and Cystatin C

**Figure 3.**
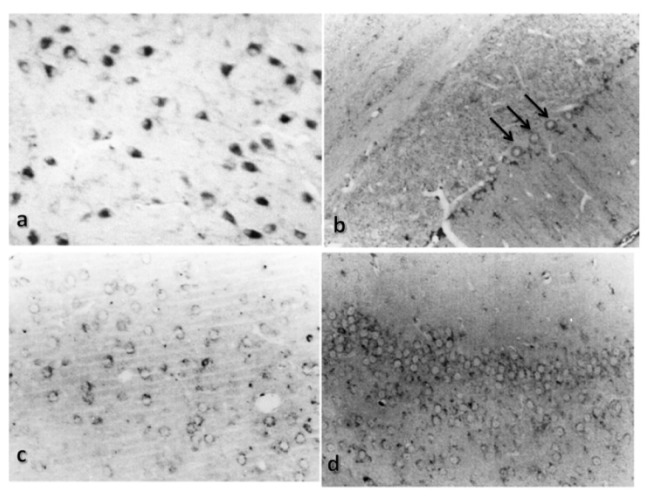
Immunolocalization of cathepsin B in control rats. Representative micrographs of (**a**) the trapezoid body, 20×; (**b**) the cerebellum, 20×; (**c**) the cerebral cortex, 20× and (**d**) the hippocampus, 20×, in a control rat. Arrows indicate cathepsin B staining.

**Figure 4.**
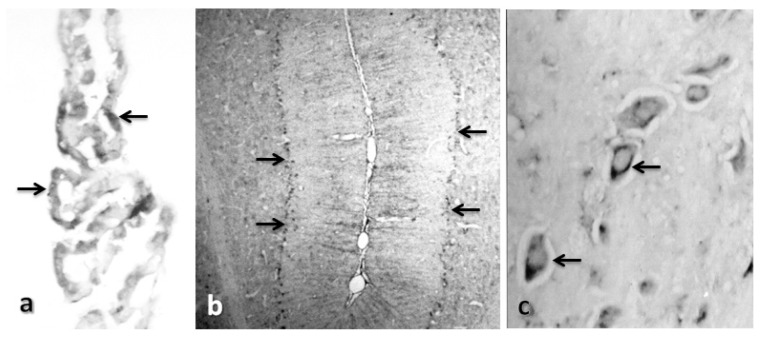
Immunolocalization of cystatin C in control rats. Representative micrographs of (**a**) the choroid plexus, 40×; (**b**) the cerebellum, 10×; and (**c**) the trapezoid body 40×, in a control rat. Arrows indicate cystatin C staining.

Cathepsin B was immunohistochemically localized in rat brain slices, predominantly in the brain stem and in the cerebellum; it was also detected in cerebral cortex and hippocampus, but with a lower intensity (see Figure 3) as previously described in the literature [20,21]. These data also agree with the mRNA distribution for cathepsin B [22]. Nevertheless, we found a little staining for cathepsin B in the hippocampus and cerebral cortex. Cystatin C was detected in the choroid plexus, pontine nuclei, the Purkinje cells and the granular and molecular layers of the cerebellum (see Figure 4). It was not detected in either the hippocampus or the cerebral cortex of control rats, which is in agreement with previous reports. Deng *et al.* [23] found that staining for CC was absent or minimal in the brains of human control subjects, whereas Lignelid *et al.* [24] found that immunoreactive CC was present in the choroid plexus epithelial cells, cerebral and cerebellar neurons in human brains. Yashura *et al.* [25] described positive staining for CC in rat brains in a few astrocytes, rare cortical neurons and the choroidal plexus. They also found a dense band of positive granules in the cerebellar cortex and in the Purkinje cell layer, which agrees with our results.

### 2.3. Effect of TBI on Cathepsin B and Cystatin C Distribution

We observed changes in the expression and localization, particularly in the cerebral cortex, brain stem and hippocampus, of both cathepsin B and cystatin C in the brains of rats after TBI.

**Figure 5.**
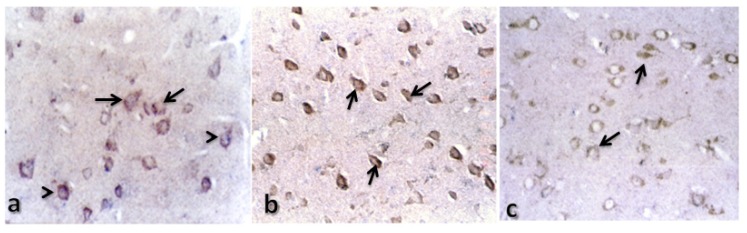
Effect of TBI in immunoreactivity of cathepsin B and CC in motor cortex. Representative micrographs showing the changes in the immunoreactivity of cathepsin B (brown) and CC (purple) of rats killed at 24 (**a**), 48 (**b**) or 72 (**c**) h after TBI. Cathepsin B was detected in the brains of control rats, and the expression level increased 24 h after TBI and decreased after 72 h. CC was only detected after TBI. Magnification: 20×. Arrows indicate cathepsin B staining, and arrowheads indicate cystatin C staining.

**Figure 6.**
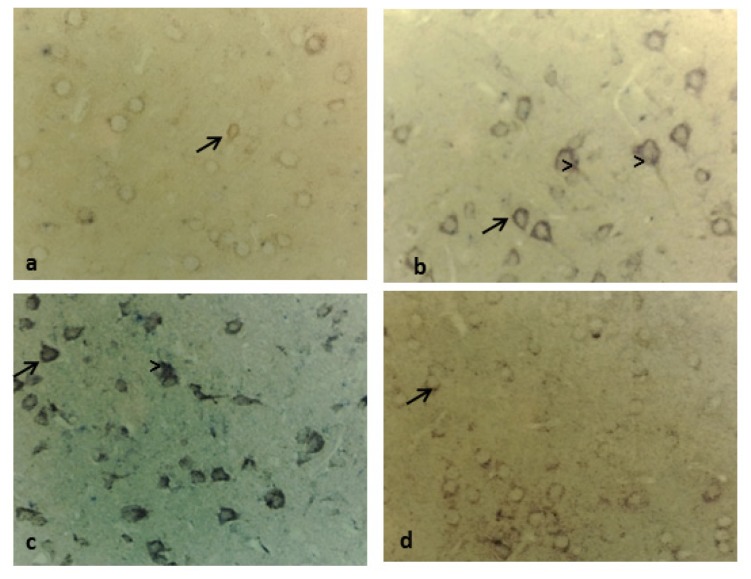
Effect of TBI on the immunoreactivity of cathepsin B and CC in the retrosplenial agranular cortex. Representative micrographs showing the change in immunoreactivity of cathepsin B (brown) and CC (purple) in rats killed either before TBI (**a**) or at 24 (**b**), 48 (**c**) or 72 (**d**) h after TBI. Cathepsin B was detected in the brains of control rats, and the expression level increased 24 h after TBI and decreased after 72 h, whereas CC was only detected after TBI. Magnification: 20× (**a**,**c**,**d**); 40× (**b**). Arrows indicate cathepsin B staining, and arrowheads indicate cystatin C staining.

Cerebral cortex. We observed changes in both the retrosplenial agranular cortex and the motor cortex. In both regions, cathepsin B was localized to the perinuclear zone of some cells in the control rats, and CC was absent. Twenty four hours after TBI, the expression level of cathepsin B increased, and it co-localized with CC. The expression levels of both proteins increased 48 h after TBI and decreased after 72 h (see Figures 5 and 6).

**Figure 7.**
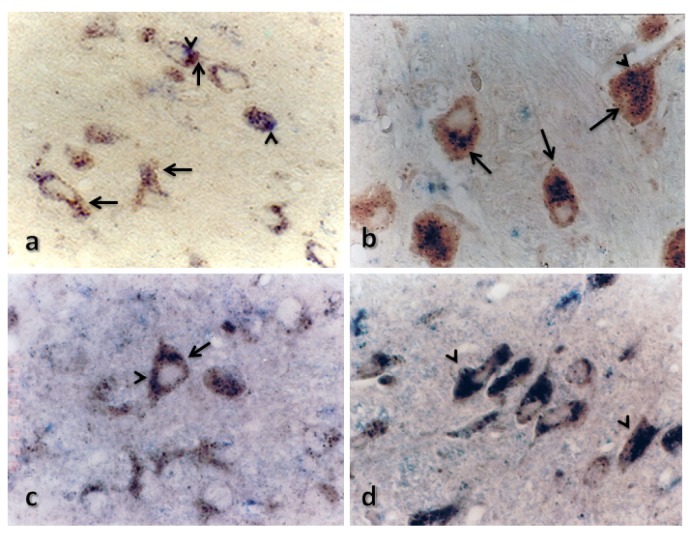
Effect of TBI on the immunoreactivity of cathepsin B and CC in the pontine nuclei. Representative micrographs showing the change in the immunoreactivity of cathepsin B (brown) and CC (purple) in rats killed either before TBI (**a**) or at 24 (**b**), 48 (**c**) or 72 (**d**) h after TBI. Both proteins were detected in control rats, and the expression levels increased after 24 h and are sustained at 72 h after TBI. Magnification: 100×. Arrows indicate cathepsin B staining, and arrowheads indicate cystatin C staining.

In the pontine nuclei, both cathepsin B and cystatin C were expressed in control rats. Forty-eight hours after TBI, cathepsin seemed to lose its lysosomal distribution and was localized to the cytoplasm, together with the CC (see Figure 7).

**Figure 8.**
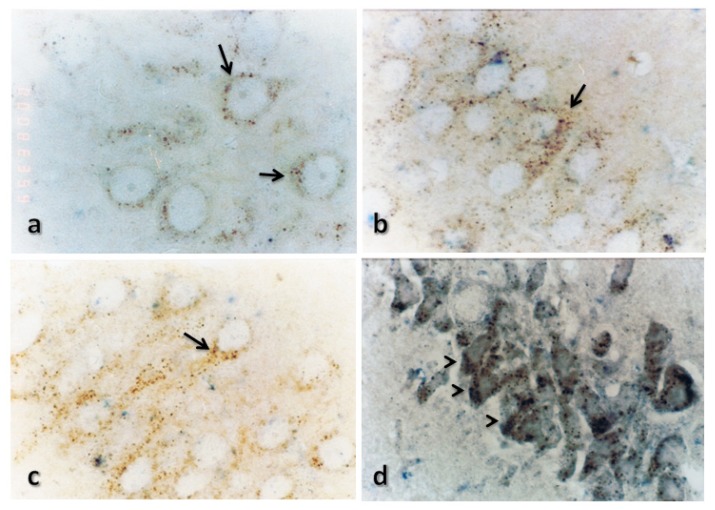
Effect of TBI on the immunoreactivity of cathepsin B and CC in CA1 region of hippocampus. Representative micrographs showing the change in the immunoreactivity of cathepsin B (brown) and CC (purple) in rats killed either before TBI (**a**) or at 24 (**b**), 48 (**c**) or 72 (**d**) h after TBI. Cathepsin B was detected in control rats, and the expression level increased 24 h after TBI and decreased after 72 h, whereas CC was detected 48 h after TBI. Damaged cells show an intense immunoreactivity for cathepsin B, which has lost its lysosomal distribution. Magnification: 100× Arrows indicate cathepsin B staining, and arrowheads indicate cystatin C staining.

**Figure 9.**
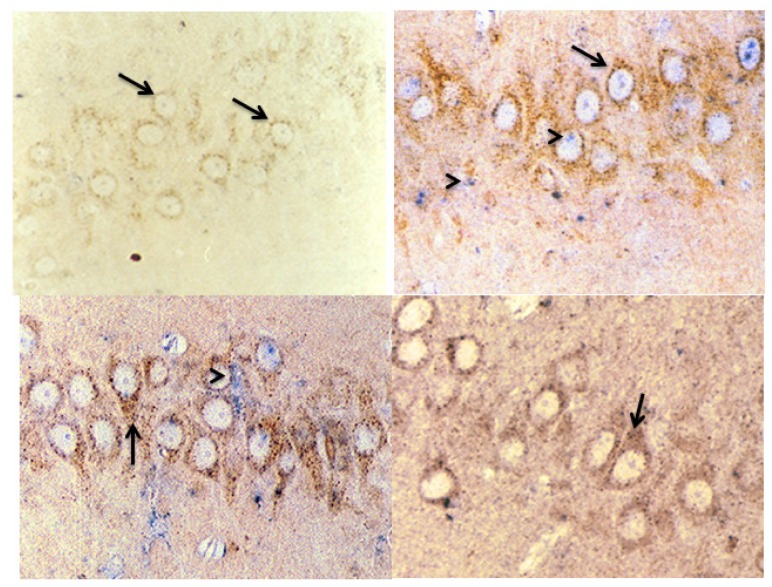
Effect of TBI on the immunoreactivity of cathepsin B and CC in the CA3 region of the hippocampus. Representative micrographs showing the change in the immunoreactivity of cathepsin B (brown) and CC (purple) in rats killed either before TBI (**a**) or at 24 (**b**), 48 (**c**) or 72 (**d**) h after TBI. Cathepsin B was detected in control rats, and the expression level increased 24 h after TBI and decreased after 72 h, whereas CC was detected 24 h after TBI. Magnification: 100×. Arrows indicate cathepsin B staining, and arrowheads indicate cystatin C staining.

Hippocampus. Cathepsin B was expressed in both the CA1 and CA3 regions of the hippocampus in control rats. Its expression level increased after TBI, and it lost its lysosomal distribution in the CA1 region 72 h after TBI. Cystatin C expression was perceptible 24 h after TBI in the CA3 region and 48 h after TBI in the CA1 region (see Figures 8 and 9).

These data agree with reports of increased cathepsin B expression in rat brains after reperfusion following a transient middle cerebral artery occlusion [26] or a global occlusion [27].

An increase in cathepsin B expression in hippocampus after transient ischemia has been reported, particularly in the CA1 neurons where cathepsin changed its lysosomal pattern to a more intense labeling, redistributed to the cytoplasm [28–30].

CC immunostaining in the cerebral cortex and hippocampus could only be detected after TBI. Other authors have reported an increase of CC in posttraumatic cerebral tissues: Palm *et al.* [31] reported a minimum CC immunostaining in control rats, but clearly visible in degenerating cells in the hippocampal CA1 region of rats subjected to ischemia. Ishimaru *et al.* [32] found an increase of CC immunostaining in the CA1 region in the gerbil hippocampus after transient ischemia. Nevertheless, they reported the presence of CC in the CA1 region of the hippocampus in control gerbils, which showed a biphasic change after ischemia: a decrease one day later and an increase by the fourth day. Similar results have been reported by Pirttilä and Pitkänen [33] using a photothrombosis model to induce epilepsy in rats.

Several authors have associated increased cathepsin B expression after transient ischemia with neuronal damage [26,28,34], whereas increased cystatin C expression was not associated with neurodegeneration [35]. In fact, we found an association between early increases in CC expression and lesser levels of neuronal damage. For example, in the CA3 subfield of the hippocampus, we observed CC expression 24 h after TBI, whereas in the CA1 subfield, CC expression could only been detected 48 h after TBI. We, and others have found that the CA1 subfield is more susceptible to damage than the CA3 subfield [36].

## 3. Experimental Section

### 3.1. Animals

Male Wistar rats (250 to 300 g) were maintained under a controlled dark-light cycle (12 h:12 h, lights on at 08:00 h) with food and water *ad libitum.* All animal experiments were performed according to institutional guidelines and were approved by the Ethics Committee of the School of Medicine

### 3.2. Intracerebroventricular Administration of CC and Traumatic Brain Injury

A stainless steel cannula (23 gauge) was stereotactically implanted into the lateral ventricle according to methods of Paxinos and Watson Atlas [37] (*p* = 0.8, *L* = 1.5, *V* = −3.8). The entire procedure was practiced under the effects of anesthetics (a mixture of ketamine, 66 mg/kg; xylazine, 0.26 mg/kg; and acepromazine, 1.3 mg/kg). After eight days of recovery, rats were housed individually, a measured amount of food was delivered, and their weight was recorded. Rats were divided into five groups: Group 1 was administered intracerebroventricularly with 4 μL of saline (control group; *n* = 36), and a CC concentration of 3.5 (*n* = 6), 35 (*n* = 7), 175 (*n* = 8) or 350 (*n* = 29) fmoles/4 μL was administered to groups 2 to 5, respectively. Fifteen minutes after the administration of saline or CC, the rats were anesthetized with chloral hydrate 6% (400 mg/kg) and traumatized. A moderate head injury was produced by dropping a weight (90 g × 50 cm) onto the intact skull at *p* = 4. This model is known as a closed head injury. A moderate-head injury was defined as an injury resulting in a mortality rate of less than 20%. For all the subjects, TBI was performed at 13:00 h.

After causing the TBI, the following characteristics were evaluated: bleeding, food and water intake for 24 h, neurological damage and mortality.

### 3.3. Bleeding

We evaluated the external hemorrhage produced by weighing the blood drained after producing the TBI. In brief, 15 min after the TBI, blood was drained and collected by pipette and then deposited into microtubes and weighed as previously described [11].

### 3.4. Neurological Damage

We used a 21-point behavioral–neurological scale [38] to evaluate the neurological damage 24 h after the TBI. We evaluated paw placement (four points), righting reflex (1), horizontal bar equilibrium (3), slanting platform (3), rotation (2), visual fore-paw reaching (2), contra-lateral reflex (2), motility (2) and general condition (2). The maximum score (minimum damage) = 21.

### 3.5. Cannula Position

Eight days after the TBI, rats were anesthetized (sodium pentobarbital 100 mg/kg ip), and perfused with 4% paraformaldehyde, and the brains were removed, frozen and sectioned (thickness: 30 μm) in a cryostat. The brain sections were collected serially from bregma −0.92 to −5.8 at 300 μm intervals from the injured area and stained with cresyl-violet to verify the position of the cannula. Only animals with the cannula at the lateral ventricle were included in the data analysis.

### 3.6. Tissue Dissection and Preparation

Another group of 12 rats were used for the following studies. Three rats were used for analyzing the basal distribution of cathepsin B and CC, and the rest were anesthetized (chloral hydrate 6%, 400 mg/kg) and subjected to the TBI. Of these, three were killed 24 h after TBI, three were killed 48 h after TBI, and three were killed 72 h after TBI. All the rats were deeply anaesthetized with sodium pentobarbital (100 mg/kg ip) and perfused transcardially with 200 mL 0.9% saline phosphate buffer (pH 7.4) followed by 200 mL 4% paraformaldehyde in 0.1 M phosphate buffer (pH 7.4). The brains were removed and post-fixed overnight in paraformaldehyde fixative at 4 °C and then were dehydrated in increasing ethanol concentrations overnight and embedded in paraffin wax.

### 3.7. Immunohistochemistry

The paraffin-embedded brains were cut into serial 6-μm thick sagittal slices, which were dewaxed and rehydrated according to standard methods. All sections were incubated in both 10 mM sodium citrate pH 6.0 for 3 min at 120 °C to retrieve antigens and in 3% H_2_O_2_ for 10 min at room temperature to quench endogenous peroxidase.

Sections were incubated with Blotto (M-7409 Sigma, St. Louis, MO, USA) for 60 min at room temperature to block non-specific binding.

For cathepsin B detection, incubation with a primary antibody was performed overnight at 4 °C with 2 μg/mL goat anti-cathepsin B (Santa Cruz Biotecnology sc-6493, Santa Cruz, CA, USA). The primary antibody was detected by incubating with 5 μg/mL biotinylated rabbit anti-goat IgG (Zymed 81-1640, Camarillo, CA, USA) for 60 min at 37 °C, followed by an incubation with 2.5 μg/mL streptavidin-biotin-peroxidase complex (Zymed 43-4323) for 60 min at 37 °C. The immunoreaction was visualized using 0.015% H_2_O_2_ in 3,3-diaminobenzidine-tetrahydrochloride (DAB Zymed kit 00-2020) as chromogen for 10 min atroom temperature.

For CC detection, the slices were incubated overnight with 10 μg/mL rabbit anti-cystatin C (Upstate biotechnology 06-458, Lake Placid, NY, USA). A goat anti-rabbit IgG–alkaline phosphatase antibody diluted 1:50 and BCIP (Zymed 00-2211) were used to detect anti-cystatin C.

For double staining, the procedure for cathepsin B detection was followed by the procedure for CC detection.

As negative controls, we performed immunohistochemistry after the above-mentioned protocols, omitting either the primary or secondary antibodies in parallel with the standard procedure.

## 4. Conclusions

We found that cystatin C administration caused a dual response in post-traumatic brain injury recovery. Higher doses increased damage, whereas lower doses decreased it. We also found that an early increase and intralysosomal expression of cystatin C after brain injury was associated with reduced neuronal damage. In conclusion, we believe that a fine balance between the cathepsins and Cystatin C must be maintained to achieve effective neuroprotection.
